# A Meta-Analysis of Osteosarcoma Outcomes in the Modern Medical Era

**DOI:** 10.1155/2012/704872

**Published:** 2012-03-18

**Authors:** Daniel C. Allison, Scott C. Carney, Elke R. Ahlmann, Andrew Hendifar, Sant Chawla, Alex Fedenko, Constance Angeles, Lawrence R. Menendez

**Affiliations:** Division of Orthopedic Oncology, Department of Orthopedic Surgery, Keck School of Medicine, Los Angeles County Medical Center, University of Southern California, 1520 San Pablo Street, Suite 2000, Los Angeles, CA 90033, USA

## Abstract

Four decades ago, specialized chemotherapy regimens turned osteosarcoma, once considered a uniformly fatal disease, into a disease in which a majority of patients survive. Though significant survival gains were made from the 1960s to the 1980s, further outcome improvements appear to have plateaued. This study aims to comprehensively review all significant, published data regarding osteosarcoma and outcome in the modern medical era in order to gauge treatment progress. Our results indicate that published survival improved dramatically from 1960s to 1980s and then leveled, or in some measures decreased. Recurrence rates decreased in the 1970s and then leveled. In contrast, published limb salvage rates have increased significantly every recent decade until the present. Though significant gains have been made in the past, no improvement in published osteosarcoma survival has been seen since 1980, highlighting the importance of a new strategy in the systemic management of this still very lethal condition.

## 1. Introduction

Osteosarcoma was once considered such a fatal condition that early studies measured outcome in terms of “months to metastasis” rather than actual survival. Shortly after the turn of the last century, Coventry measured osteosarcoma survival at 5% [1]. In the 1950s, neither surgery, nor radiation, nor rudimentary chemotherapy regimens significantly impacted survival, with the largest study of the decade citing a 22% 5-year survival [1]. With the advent of higher dose, multiagent chemotherapy regimens, 5-year survival steadily increased to as high as 81.6% in the 1970s [2].

However, casual inspection of published data indicates that since the 1970s, survival and perhaps other outcome measures have yet to further improve. As surgical techniques and implants have evolved, chemotherapeutic agents used today seem to be wholly similar to those used thirty years ago.

A meta-analysis of the world's data regarding osteosarcoma and outcomes would help us accurately gauge our current state of treatment and not just speculate on improvements or stasis based on isolated recent studies and anecdotal evidence. Several studies have attempted similar meta-analyses; however, their scales were much smaller (the largest of which contained 142 series) and their scopes were narrower (focusing only on specific therapies) than our intended breadth [3–5]. The purpose of our study is to thoroughly inspect the expansive literature regarding prognosis of osteosarcoma since the inception of journal publication, in order to analyze the treatment trends across the globe in regards to time. Thus, we aim to determine treatment progress, as well as the current state of affairs, so that we may in turn accurately gauge our progress and appropriately direct our efforts.

## 2. Materials and Methods

A comprehensive and complete MEDLINE search was performed, using only the search terms “osteosarcoma” and “survival.” Articles not written or translated into English, animal or in vitro studies, and articles not accessible through the University of Southern California Norris Medical Library were excluded. All articles describing outcome data were included, including clinical trials, case series, databases, letters, and reviews. General case series with less than 20 total patients were excluded. All studies including nonclassic or lower grade osteosarcoma variants as well as secondary osteosarcoma or recurrent osteosarcoma were excluded, as were head and neck cases. Pelvic and axial tumors were included. Studies that measured survival in terms of odd years (e.g., 7-year survival) and, for studies published after 1980, those series which measured outcomes in less than 5-year terms were also excluded. Metastatic series were included, however, they were left apart from the general studies and analyzed separately.

Both overall survival (OS) and disease-free survival (DFS) of 5 years or more were recorded from each study, focusing on 5- and 10-year survival numbers when possible. Local recurrence and limb salvage rates were also measured, if data was given. Each study was then assigned to specific time intervals (decades), which were determined by the described study period of each series. If a series spanned two decades, it was placed in the decade in which a majority of the study time occurred, rounding to the more recent decade if the division was equal. If a series spanned more than two decades, it was placed in a separate table for direct review (Table 1), and not included in the decade-to-decade analysis. If the article did not mention the time period, the study was placed into the decade preceding that of publication. If a study was divided according to decade or time period, each arm was placed into its separate decade category accordingly.

Of 3,948 initial series found in the search for “osteosarcoma” and “survival,” 3,684 did not meet the study inclusion criteria, leaving a total of 264 series in the study. Data from these series were then combined in weighted fashion (according to the number of patients in each study) in order to obtain mean values for each respective outcome measure. Nonmetastatic cases were evaluated in terms of survival (OS (5, 10 year) and DFS (3, 5, and 10 year)) recurrence, and limb salvage rates, and analyzed according to decade. Articles that recorded specific subtypes (metastatic cases, pelvis, and limb salvage) were also analyzed separately according to decade with regard to 5-year OS. The data from metastatic series were not included in the general survival, limb salvage, and recurrence data. Along each outcome measure, these means were then compared according to the *t*-test for proportions, set to a 95% confidence interval.

## 3. Results

### 3.1. General Survival

**Figure 1:**
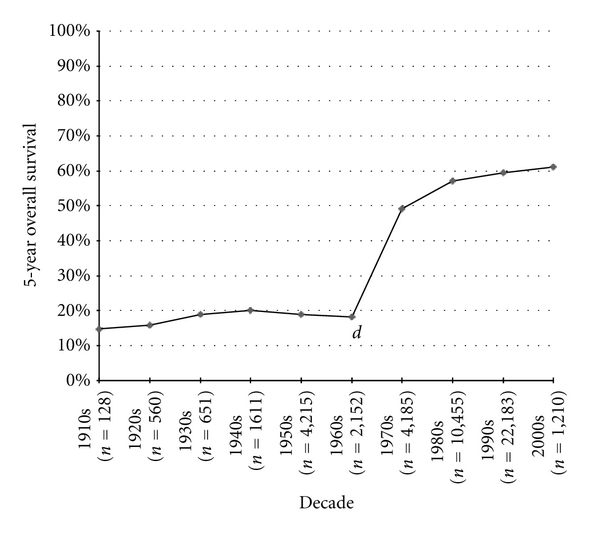
Osteosarcoma 5-year overall survival.

**Figure 2:**
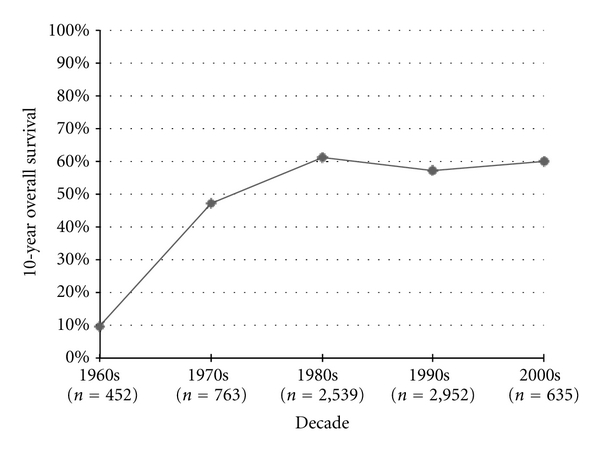
Osteosarcoma 10-year overall survival.

Among the series of nonmetastatic, high-grade osteosarcoma patients, the most commonly measured statistic, 5-year OS was measured in 47,227 patients throughout the series.  Figure 1 shows the 5-year OS trend from the beginning of the twentieth century to present day. Survival remained stable at approximately 20% from the 1910s to the 1960s, with a sudden surge to approximately 60% into the 1980s where it stabilized again. The increase in 5-year OS from the 1960s to the 1970s (*P* < 0.0001) and from the 1970s to the 1980s (*P* < 0.0001) was statistically significant; however, when comparing the 1980s to the 2000s, no further statistically significant increase in published 5-year OS was recorded (*P* = 0.66). Ten-year OS increased by 37.6% from the 1960s to the 1970s (*P* < 0.0001), but then showed no difference when comparing the 2000s to the 1990s (*P* = 0.28) (Figure 2).

**Figure 3:**
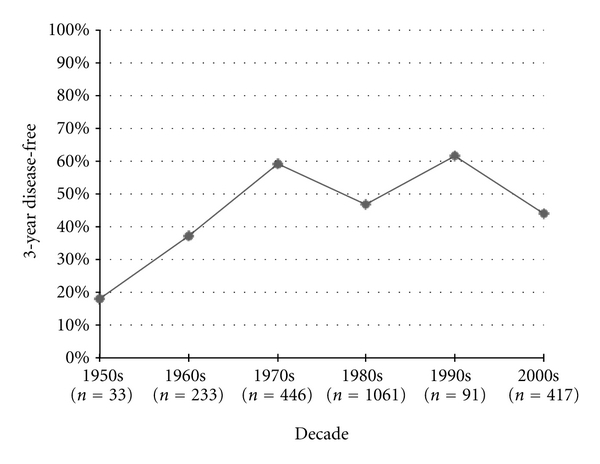
Osteosarcoma 3-year disease-free survival.

**Figure 4:**
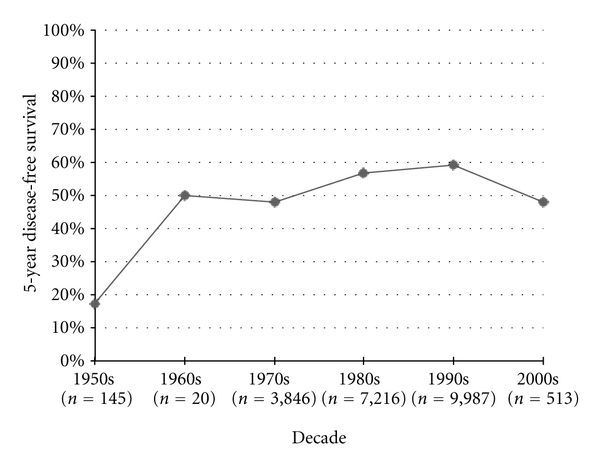
Osteosarcoma 5-year disease-free durvival.

**Figure 5:**
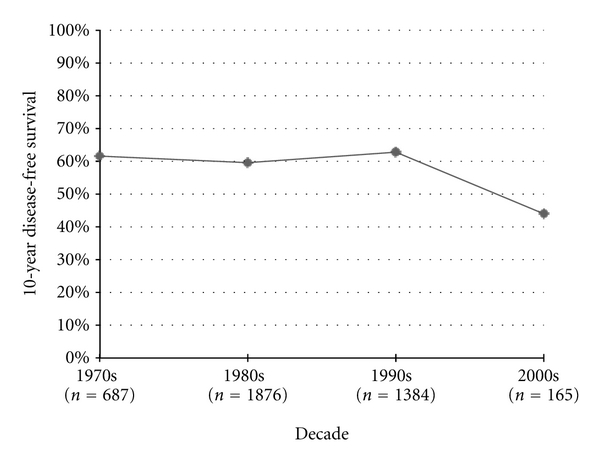
Osteosarcoma 10-year disease-free survival.

Disease-free survival was first recorded in the 1950s studies, starting with 3-year DFS measures. Three-year DFS increased significantly from the 1950s to the 1960s (*P* = 0.05) and from the 1960s to the 1970s (*P* < 0.0001), but then failed to improve after the 1970s (Figure 3). The published 5-year DFS increased by 8.6% from the 1970s to the 1980s (*P* < 0.0001) and 2.6% from the 1980s to the 1990s (*P* = 0.0007), but then significantly decreased by 11.2% from 1990 to 2000 (*P* < 0.0001) (Figure 4). Similarly 10-year DFS remained stable in the 1970s, 1980s, and 1990s at roughly 60%, only to statistically decrease to 44% in the 2000s (*P* < 0.0001) (Figure 5).

### 3.2. Local Control and Limb Salvage

**Figure 6:**
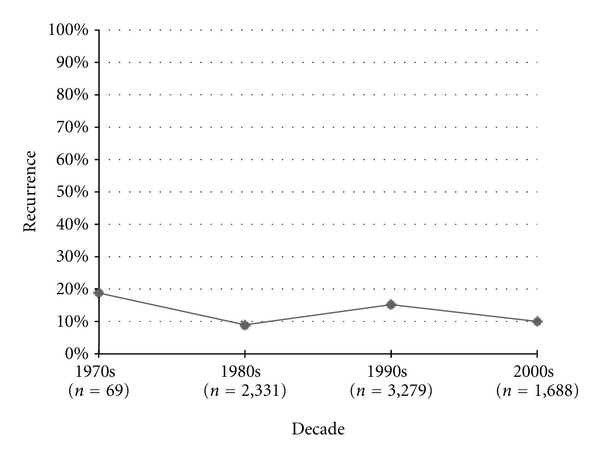
Osteosarcoma recurrence rates.

Recurrence measures were initially published in the 1970s, and have fluctuated from decade to decade since (Figure 6). No significant improvement has been seen in published recurrence rates from the 1980s to the 2000s (*P* = 0.36).

**Figure 7:**
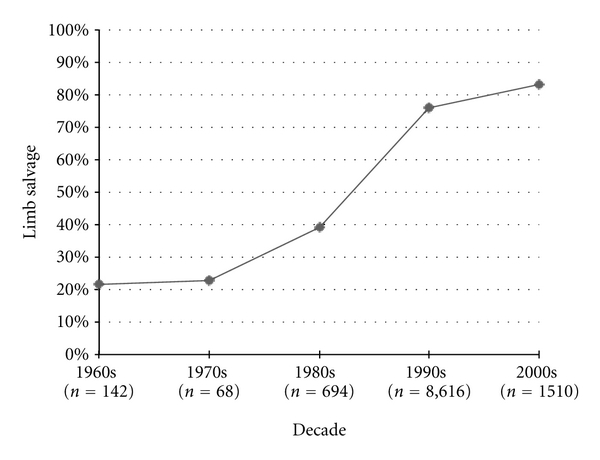
Osteosarcoma limb salvage rates.

Limb salvage rates have improved significantly from decade to decade ever since the 1970s, improving by 16.2% from the 1970s to 1980s (*P* = 0.01), by 37% from the 1980s to the 1990s (*P* < 0.0001), and by 7.3% from the 1990s to the 2000's (*P* < 0.0001) (Figure 7).

### 3.3. Survival in Specific Osteosarcoma Populations

**Figure 8:**
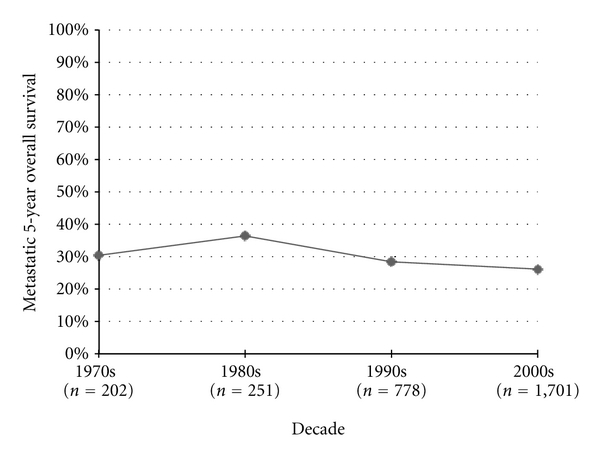
Metastatic osteosarcoma 5-year overall survival.

Starting in the 1970's, cases of metastatic osteosarcoma were analyzed in separate series. Five-year survival rates in metastatic series improved from the 1970's to the 1980's by 5.7%, though this change was not statistically significant. Interestingly, 5-year survival rates in this population have decreased every decade ever since, with no statistical change in survival when comparing the 2000's to the 1970's (*P* = 0.21) (Figure 8).

**Figure 9:**
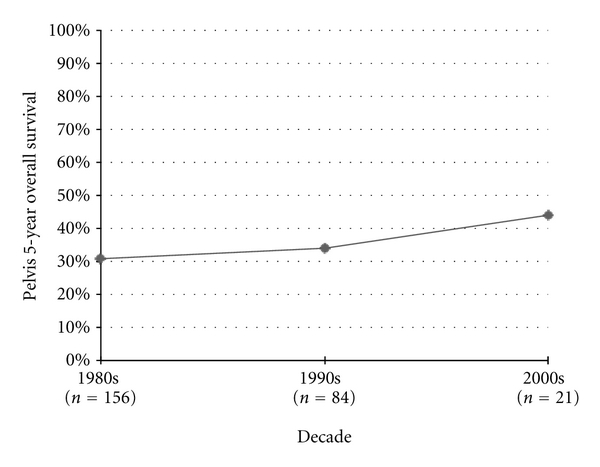
Pelvic osteosarcoma 5-year overall survival.

When looking specifically at series of pelvic osteosarcoma patients, survival rates have also declined every decade since their first recording in the 1980's; however these drops were not statistically significant (Figure 9).

**Figure 10:**
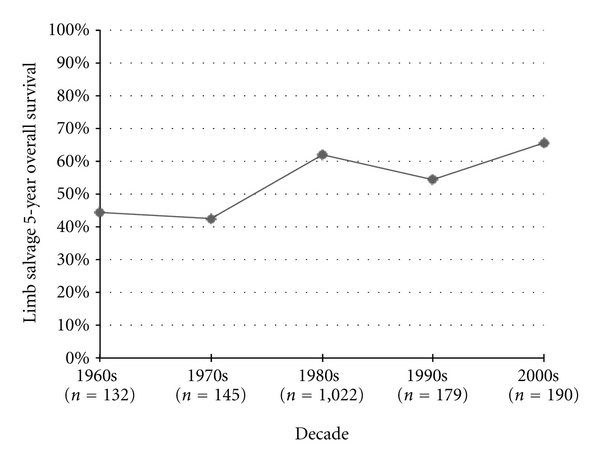
Osteosarcoma 5-year overall survival among limb salvage cases.

In specifically documented limb salvage series, 5-year OS rates improved significantly from the 1990's to the 2000's by 11.1% (*P* = 0.04) and from 1970's to the 2000's by 23.1% (*P* < 0.0001) (Figure 10).

## 4. Discussion

Advances in chemotherapy regimens in the seventies lead to extraordinary increases in survival of osteosarcoma patients which, combined with improved surgical technology and technique, also lead to significant improvements in limb salvage. Physicians anecdotally refer to the lack of survival progress in the latter end of the last century, yet few studies probe into the extensive world literature. The few that attempt to address this issue narrow their reviews to controllable numbers: as few as 8 in one review [5]. Anninga et al. recently published the previously most comprehensive review, but looked at specific treatment regimens and excluded other prognostic, observational, and review studies [3]. We aimed to sort through the extensive osteosarcoma literature to arrive at basic evidence-based interpretations regarding the current status of osteosarcoma management, as it relates to our past. Such information will help us determine the most appropriate steps to take as an osteosarcoma-treating community as we progress into the new century.

Despite its expansive scale, our study certainly has important limitations. Our numbers are based on published studies, which may not accurately represent outcomes in the community as a whole. Many successive studies may contain duplicate patients, skewing the data in this direction. According to our exclusion criteria, we may have excluded important contributing data, especially with the removal of studies with odd-year survival measures. Nevertheless, the USC Norris Medical Library is extensively complete, and the exclusion criteria narrow, decreasing the likelihood of missing significant data. Furthermore, our retrieved number of studies was so high that the addition of this data is very unlikely to change any calculation. Furthermore, the analysis of the extensive amount of data could pose limitations. The homogenous blending of different survival curves from heterogenous populations could skew outcome results. The incorporation of numerous studies with large numbers of patients helps mitigate this risk for error. Not all of the predominately survival studies gave data on recurrence or limb salvage rates. These numbers were weighted averaged according to decade; however the exact number and subsequent power of their information is less than that of survival; however, even with these lower numbers, the numbers were still high enough to note significant differences. Combining all osteosarcoma patient populations together may also skew results, as certain patient populations may have different outcomes (i.e., axial versus appendicular cases). In order to address this limitation, we analyzed specific population series separately in order to show their survival data (Figures 8, 9, and 10). Combining single center and multicenter studies also poses a limitation, increasing the associated variables such as treatment regimens and outcome measures. Again, the very high numbers retrieved in our study (47,227 patients in the 5 year overall survival data) help mitigate these limitations.

Our study confirms suspicions regarding the lack of statistical improvement in osteosarcoma survival over the last thirty years. In fact, DFS at the 3-year, 5-year, and 10-year marks have shown recent decreases over the last two decades. After steep improvements up until the 1970's, overall survival at the 5 and 10-year marks has simply plateaued with lack of statistical improvements. Similarly, recurrence rates have fluctuated in the modern era, without significant improvement. This lack of improvement is also true in subset populations like pelvic metastatic cases, with actual decreases in survival in both of these populations over the last two decades, though these decreases did not reach statistical significance. Of note, limb salvage rates as well as survival rates in limb salvage populations, have continued to climb, increasing significantly since the 1970's on both accounts. This progress may indicate improvements in biopsy, respective techniques, and limb salvage reconstructive methods.

In a time of unprecedented technological growth and advance, systemic cancer treatment clearly lags behind. This lag is further accentuated by the tremendous surge in survival of osteosarcoma until the 1980's. Our study indicates that this stagnation is not limited to a single outcome measure, but across all outcomes ranging from survival to recurrence.

After noting the problem, our next charge is to find the underlying cause. Given the expense of drug development, trialing, and now even marketing, the relatively low numbers associated with osteosarcoma, especially in relation to visceral malignancy, renders the orphan condition less popular among pharmaceutical companies that have an obligation to shareholders. Furthermore, the lack of profit from cure of disease as opposed to prolongation of disease likely lessens the industry's quest for a cure. Unfortunately, in the current system, economic incentives do not always align with patient wellness incentives. A similar problem was addressed with osteosarcoma in the early 1970's with the touching story of Terry Fox, which highlighted the lack of efforts put into osteosarcoma research and drug development. The patient's story, which became a feature film, helped spawn an unprecedented interest and motivation in curing the disease worldwide. Perhaps efforts to show the public the plights of the often young patients struck with this disease will lead to a second surge in interest. Another potential cause of this lack of progress could be complacency among those who treat sarcomas. While many oncologists have cited the lack of progress in the treatment of non-sarcoma cancers in the past, opponents would cite the survival jump of osteosarcoma as a sign of cancer fighting progress. The idea that osteosarcoma is a bastion of chemotherapeutic advance may have decreased the scientific sense of urgency in finding a cure.

Regardless of the underlying cause, which may be very difficult to ever determine, a change in strategy must be employed if we are to change our results. The paradigm shift from the use of cytotoxic agents to molecularly targeted agents may be a source of positive change. The theory of tumor stem cells explaining tumor behavior is an example of potential fundamental changes in the way we understand these tumors, which lead to fundamental changes in treatment. Using the gains in the treatment of other conditions that gain high level industry attention (e.g., RANK-L inhibitors for skeletally related events in metastatic disease and in osteoporosis) would be another potential area for improvement. Increased media attention in the form of news outlets, publications, or even entertainment forums would direct attention and money to the cause. Empowering academic centers to research, develop, and even market themselves would bring competition into the chemotherapy marketplace and focus on outcome measures and potentials for long-term economic gain in the form of cure that shortsighted companies may not currently see. Increased regulation regarding the use of human subjects in research may be an issue, but the unfortunate, but real number of patients with metastases may serve as a pool for such studies, and thus would be the first to benefit.

## 5. Conclusions

Our study confirms suspicions about the stagnation of progress in systemic osteosarcoma management in recent decades. Limb salvage rates have continued to improve significantly throughout the modern era to the present day; however, after tremendous improvement in the 1970's, survival measures have failed to demonstrate any further increased since the 1980's. After 30 years of lack of progress, we should reevaluate our treatment paradigms and think along different lines, especially in regard to the current players behind drug and medical technology development as well as our own attitudes toward disease treatment and outcomes we deem appropriate.

## Figures and Tables

**Table 1:** Osteosarcoma outcome studies spanning more than two decades (OS: overall survival, DFS: disease free survival).

Category	Journal	Lead author	Pub year	Focus	Source dates	*n*	DFS 3 yrs	DFS 5 yrs	DFS 10 yrs	OS 5 yrs	OS 7 yrs	OS 10 yrs	Recurr-ence	Limb salvage
General	Human Path	Gaffney	2006	General	1900–1966	465				18.0%				
	Cal Med	Higinbotham	1965	General	1925–1955	130				18.5%		16.2%		
	CORR	Johnson	1971	General	1928–1966	50				10.0%			14.0%	36.0%
	Cancer	Ohno	1971	General	1930–1960	41				17.1%				
	Can J Surg	Huebert	1979	General	1930–1977	95				16.0%				
	Can J Surg	Huebert	1979	General	1930–1977	95				16.0%				
	JBJS Br	Foster	2007	General	1933–1959	31				21.0%				20.0%
	Acta Chir Scand	Poppe	1968	General	1938–1964	102				18.0%				
	J Surg Oncol	Shrikhande	1977	General	1941–1970	139				15.1%				
	J Surg Oncol	Rao	1977	General	1941–1970	139				15.1%				
	CORR	Weiss	1978	General	1944–1975	50				10.0%				
	JBJS Am	Price	1975	General	1946–1972	125		12.0%						
	Cancer	Jaffe	1972	General	1950–1972	78				17.0%				
	JBJS Am	Bleyer	1982	General	1952–1973	19				41.0%				
	CORR	Brostrom	1982	General	1952–1974	57	19.0%							
	Eur J Surg Oncol	Pylkkanen	1997	General	1958–1987	29			46.7%					
	Acta Orthop Belg	Meskens	1993	General	1962–1987	51					41.0%			
	CORR	Tunn	2004	General	1970–1997	78						70.0%		
	Eur J Cancer	Bacci	2005	General	1972–1999	114 8		57.0%	52.0%	66.0%		57.0%		
	Ped Perinat Epid	Parkes	2010	General	Pre 1978	NS						12.0%		
	Ped Perinat Epid	Parkes	2010	General	Post 1977	NS						45.0%		
	Cancer	Kager L	2010	General	1979–2004	27		48.0%		51.0%			40.7%	
	Acta Chir Scand	Poppe	1968	General	1938–1964	102				18.0%				

Age	Cancer	Mirabello	2009	All Ages	1973–2004	348 2				37.0%		32.0%		
				0–24		185 5				62.0%				
				25–59		974				58.0%				
				60+		653				24.0%				
	Pediatr Blood Cancer	Abate	2010	0–5	1972–1999	20		60.0%						
				6–40		110 6		53.8%	52.1%					
	Pediatr Blood Cancer	Worch	2010	0 – 5	1973–2006	38				51.9%				
				6–19		126 8				67.3%				
	J Cancer Res Clin Oncol	Harting	2010	0–10	1980–2000	7				50.0%		46.5%		
				10–21.		235				54.0%		46.0%		
				21–40		110				68.5%		55.0%		
				40+		56				40.0%		37.0%		
	J Chin Med Assoc	Hsieh	2009	Preadolescent	1980–2006	11				60.6%				
				Adolescent		39				66.7%				
	J Clin Oncol	Longhi	2008	65+	1961–2006	29				45.0%				
	Ann Surg Oncol	Okada	2004	50+	1972–2002	55				58.6%				
	J Pediatr Hematol Oncol	Bacci	2005	0–12	1972–1999	317		60.0%		67.0%				
				13–40		819		58.0%		65.0%				
	CORR	Carsi	2002	40+	1977–1998	47		32.5%						

Treatment	Eur J Can	Bruer	1978	Lung Irradiation	1950–1973	106				9.0%				
	Arch Orthop Trauma Surg	Kawai	1996	With Chemotherapy	1965–1993	32					47.0%			
				Without Chemotherapy		9					38.0%			
	Cancer	Gupta	2009	Proximal humerus, deltoid-sparing resection	1978–2005	23				77.0%				
	J Surg Oncol	Kim	2009	Inadvertant curettage w/o treatment delay	1985–2005	20		90.0%		89.4%				
				Control Group		40		76.8%		83.9%				
	Radiology	Phillips	1969	Preop Radiation	1945–1965	23				26.0%				
				Surgery Only		11				9.0%				
	Int J Radiat Oncol Biol Phys	DeLaney	2005	Resection	1980–2002	36		51.9%		73.9%			32.3%	
				No Surgery		5		25.0%		25.0%			60.0%	

Tumor feature	Pediatric Blood Cancer	Yaclin	2008	C-erb-2+	1976–2007	24		22.4%		32.3%				
				C-erb-2−		32		50.0%		62.1%				
	Clin Transl Oncol	Gonzalez-Bilalabeitia	2009	COSS	1977–2003	170 2			48.9%			58.9%		
				High LDH		14				43.0%				
				Normal LDH		52				65.0%				
	Pediatr Blood Cancer	Franke	2011	Local recurrence	1980–2003	38		27.0%		34.0%				
	Pediatr Blood Cancer	Lee	2009	Low Risk	1985–2006	98		81.6%	81.6%	93.8%		93.8%		
				Intermediate Risk		128		59.0%	57.8%	76.5%		74.6%		
				HighRisk		62		34.0%	31.6%	47.0%		36.5%		
	Eur J Cancer	Grimer	2005	Local Recurrence	1976–2001	57				41.0%				

Location	Canc Treat Res	Bielack	2009	Extremity	1980–2005	246 4				75.0%		70.0%		
	JBJS Br	Grimer	1999	Pelvis	1971–1996	36			18.0%					
	Spine J	Schoenfeld	2010	Spine	1982–2008	26				31.0%				
	Jpn J Clin Oncol	Lee	2010	Flat Bone	1985–2006	17		39.6%		51.5%				
				Extremity		424		61.4%		72.9%				
	Eur J Cancer Care	Shenoy	2008	Humerus	1950–1975	18			35.0%					
				All Sites		112 6		55.0%		66.0%				
	CORR	Schneidrbauer	2007	Fibula	1919–2000	36				53.0%		41.0%		
	Ann Surg Oncol	Daecke	2005	Hand and Forearm	1977–2000	33		65.4%		86.2%				
	Ned Tijdschr Geneeskd	Ham	2000	Pelvis	1957–1995	62				15.0%				
	Oncology	Matsuo	2005	Pelvis	NS	50				29.5%				

Metastatic	J Clin Oncol	Longhi	2008	Age >65 yrs	1961–2006	14				0.0%				
	Ann Surg Oncol	Okada	2004	Age >50 yrs	1972–2002	7				25.0%				
